# Recombinant anti-Müllerian hormone in the maturation medium improves the in vitro maturation of human immature (GV) oocytes after controlled ovarian hormonal stimulation

**DOI:** 10.1186/s12958-022-00895-5

**Published:** 2022-01-24

**Authors:** Jure Bedenk, Tadeja Režen, Taja Železnik Ramuta, Nina Jančar, Eda Vrtačnik Bokal, Ksenija Geršak, Irma Virant Klun

**Affiliations:** 1 Clinical Research Centre, University Medical Centre Ljubljana, 1000 Ljubljana, Slovenia; 2 Institute of Biochemistry and Molecular Genetics, Centre for Functional Genomics and Bio-Chips, Faculty of Medicine, University of Ljubljana, 1000 Ljubljana, Slovenia; 3 Institute of Cell Biology, Faculty of Medicine, University of Ljubljana, 1000 Ljubljana, Slovenia; 4 Department of Gynaecology and Obstetrics, University Medical Centre Ljubljana, 1000 Ljubljana, Slovenia; 5 Faculty of Medicine, University of Ljubljana, 1000 Ljubljana, Slovenia

**Keywords:** Anti-Müllerian hormone, Anti-Müllerian hormone receptor 2, In vitro maturation, Human oocyte, Immunocytochemistry, Confocal microscopy, RT–qPCR

## Abstract

**Background:**

In vitro maturation (IVM) of oocytes is a laboratory method that allows the maturation of immature (GV) oocytes retrieved from patients enrolled in the in vitro fertilization (IVF) programme. However, this method is still sparsely researched and used in clinical practice, leading to suboptimal clinical results. Anti-Müllerian hormone (AMH) is an important hormone with known effects on human ovaries, especially on follicles (follicular cells) during folliculogenesis. In contrast, the effect of AMH on the human oocyte itself is unknown. Therefore, we wanted to determine whether human oocytes express AMH receptor 2 (AMHR2) for this hormone. Recombinant AMH was added to the IVM medium to determine whether it affected oocyte maturation.

**Methods:**

In total, 247 human oocytes (171 immature and 76 mature) were collected from patients enrolled in the intracytoplasmic sperm injection (ICSI) programme who were aged 20 to 43 years and underwent a short antagonist protocol of ovarian stimulation. The expression of AMHR2 protein and *AMHR2* gene was analysed in immature and mature oocytes. Additionally, maturation of GV oocytes was performed in vitro in different maturation media with or without added AMH to evaluate the effect of AMH on the oocyte maturation rate.

**Results:**

Immunocytochemistry and confocal microscopy revealed that AMHR2 protein is expressed in both immature and mature human oocytes. AMHR2 was expressed in a spotted pattern throughout the whole oocyte. The IVM procedure revealed that AMH in maturation medium improved GV oocyte maturation in vitro, as all oocytes were successfully matured in maturation medium containing recombinant AMH only. Furthermore, antagonism between AMH and follicle-stimulating hormone (FSH) during the maturation process was observed, with fewer oocytes maturing when both AMH and FSH were added to the maturation medium. Finally, *AMHR2* gene expression was found in immature and in vitro matured oocytes but absent in mature oocytes.

**Conclusions:**

The positive AMHR2 protein and *AMHR2* gene expression in human oocytes shows that AMH could directly act on human oocytes. This was further functionally confirmed by the IVM procedure. These findings suggest the potential clinical application of recombinant AMH to improve IVM of human oocytes in the future.

**Supplementary Information:**

The online version contains supplementary material available at 10.1186/s12958-022-00895-5.

## Background

Assisted reproductive technology (ART) has enabled many couples with fertility problems to conceive a child with the help of in vitro fertilization (IVF). Even though the technology and methods are evolving and becoming more sophisticated, there is still not much we can do regarding immature oocytes that are obtained by follicular aspiration after controlled ovarian hormonal stimulation. In most clinics, immature oocytes are discarded as part of daily practice, especially if they are at the germinal vesicle (GV; prophase I) stage, because they cannot be fertilized in the IVF programme. Fortunately, the percentage of immature oocytes obtained in the IVF programme is relatively low [1, 2]. However, for certain patients, such as those with a low oocyte count (“poor responders”) and oncology patients who undergo oocyte cryostorage for fertility preservation before chemo- and radiotherapy, these oocytes are very important and may be the only chance for embryo development and pregnancy achievement. Thus, in vitro maturation (IVM) of immature oocytes has been developed. In short, this is a laboratory method that involves collecting and culturing immature oocytes in maturation medium to obtain mature (MII) oocytes over time. These immature oocytes can be obtained from stimulated cycles after controlled ovarian hormonal stimulation, as well as from unstimulated cycles without hormonal stimulation in cancer patients, where time is of the essence and hormones pose a threat to the growth and spreading of the cancer, or in patients with polycystic ovary syndrome (PCOS), where the risk of ovarian hyperstimulation syndrome (OHSS) after hormonal stimulation is high [3, 4]). Afterwards, the oocytes are treated as mature (MII; metaphase II) oocytes and are fertilized in the IVF programme. As of 2021, IVM method is no longer treated as experimental, so it should have more clinical relevance [5]. To mature them in vitro, immature oocytes are cultured in complex maturation media with added hormones, such as follicle-stimulating hormone (FSH) and human chorionic gonadotropin (hCG), to increase the chances of their maturation [6]. The environment of the human ovary (ovarian niche) and in vivo maturation are still not fully understood (especially at the molecular level). Therefore, the human oocyte IVM procedure is not optimal at the moment, and only a relatively low percentage of oocytes manage to mature in this way [6]. Additionally, a low implantation rate and a high miscarriage rate are observed for IVM oocytes in comparison to conventional IVF of oocytes that mature in the patient’s ovaries after controlled ovarian hormonal stimulation [7].

The role of anti-Müllerian hormone (AMH) in oocyte maturation is not yet known. This dimeric glycoprotein [8, 9] is known mostly for its role in gonadal differentiation during embryonic development [10] and has an impact on ovarian function, particularly in terms of the growth and recruitment of follicles during folliculogenesis [11, 12]. It is considered to be one of the best biomarkers for the evaluation of a patient’s ovarian reserve [13–15], response to controlled ovarian stimulation [16] and the outcome of IVF, although the results of the latter vary [17, 18]. It is also predicted that AMH can become a powerful tool for the diagnosis of certain ovarian illnesses, such as PCOS [19, 20], primary ovarian insufficiency (POI) [21], premature menopause [22] and gynaecological tumours [23–25]. The key receptor for AMH is anti-Müllerian hormone receptor 2 (AMHR2), which is expressed in testicular Sertoli [26–28] and Leydig cells [28–30], ovarian theca [28, 31] and granulosa cells [26–28], as well as in the prostate [32], breasts [33] and endometrium [34]. However, we were unable to find any data regarding the expression of AMHR2 on human oocytes in the literature, in comparison to certain animals, such as mice [35] and cattle [36].

Some scarce data in animal models have shown that AMH might act directly on oocytes and affect their maturation. In 2014, Zhang et al. [35] studied the effect of AMH on the maturation of immature mouse oocytes and proved that AMH has a positive effect on oocyte maturation in vitro. To our knowledge, this is the only study to date to test the effect of AMH on oocytes, even though the study was done in mice. AMH helps to keep ovarian follicles in an inactive state so that the ovarian reserve of the woman does not diminish too early [37, 38]. In this study, we wanted to determine if the same is true for human oocytes, namely, if AMHR2 is expressed in immature and mature human oocytes and if the addition of AMH to the maturation medium affects oocyte maturation in vitro.

## Methods

### Patients and sample collection

This study included 247 (171 GV and 76 MII) oocytes from 96 patients enrolled in the IVF/intracytoplasmic sperm injection (ICSI) programme. Oocytes were retrieved from each patient after written informed consent was obtained at the University Medical Centre Ljubljana, Slovenia, from January 2019 to April 2021. The inclusion and exclusion criteria were different depending on the method used. For immunocytochemical staining, the only inclusion criteria were enrolment in IVF treatment (40 patients) and a patient age of 20 to 43 years. Patients with a low number of oocytes (< 3 total oocytes) attained by ultrasound-guided ovarian (follicular) aspiration or who were involved in the preimplantation genetic diagnosis (PGD) programme were excluded. On the other hand, for IVM and reverse-transcription quantitative real-time polymerase chain reaction (RT–qPCR) analysis, we used the following inclusion criteria: age from 20 to 38 years and ovarian stimulation using the short gonadotropin-releasing hormone (GnRH) antagonist protocol (56 patients). The exclusion criteria were the same as those for the immunocytochemical analysis.

### Controlled ovarian hormonal stimulation

Briefly, the GnRH antagonist protocol of ovarian stimulation was performed as follows: recombinant FSH stimulation was initiated on the 2nd day of the menstrual cycle. The daily dosage was adjusted individually depending on the patient status and ovarian response (between 1050 and 3000 IU; Gonal-F, Merck Europe B.V., The Netherlands or Bemfola, Gedeon Richter, Hungary or Pergoveris, Merck Europe B.V., The Netherlands). The patients were given 0.25 mg of cetrorelix acetate (Cetrotide, Merck Europe B.V., The Netherlands) or 0.25 mg of ganirelix (Orgalutran, MSD, USA) on Day 7 of the menstrual cycle, and the application continued daily until the day of the follicle trigger.

When a sufficient number of follicles reached a mean diameter of at least 17 mm, patients were administered 250 µg of recombinant hCG (Ovitrelle®, Merck Europe B.V., The Netherlands). Oocytes were obtained using transvaginal ultrasound-guided needle aspiration of follicles 34 to 36 h after the application of recombinant hCG.

### Oocyte preparation

After aspiration, cumulus-oocyte complexes (COCs) were transferred to flushing medium (Cooper Surgical, Denmark) for the removal of granulosa cells. After granulosa cell removal, oocytes were transferred to Universal IVF medium (Cooper Surgical, Denmark) and incubated for 1.5–2.5 h (37.0 °C and 6% CO_2_). Then, the oocytes were treated with hyaluronidase (SynVitro Hydase, Cooper Surgical, Denmark) for 20 s to remove the corona radiata cells. Thus, the cells were prepared for the following procedures.

### Immunocytochemical staining

For immunocytochemical staining, 65 immature and 61 mature oocytes that did not fertilize after ICSI were immunostained using a modified protocol written by Virant Klun et al. [39]. In brief, the oocytes were first washed with PBS (Gibco, USA) and then fixed in 4% paraformaldehyde (Kemika, Croatia) for 8 min. After fixation, oocytes were washed two times with phosphate-buffered saline (PBS) and permeabilized with 0.3% Triton X-100 for 10 min. Then, oocytes were washed again with PBS. Unspecific binding of antibodies was blocked by incubating oocytes in 10% foetal bovine serum (FBS) (Gibco, USA) in the dark for 20 min. The detection of AMHR2 receptors was performed with a primary monoclonal mouse anti-AMHR2 antibody (Ab64762, Abcam, USA, 1:100 dilution) for 60 min in the dark. After this, oocytes were washed three times with PBS. For the visualization of the signal, secondary goat anti-mouse antibody was used (Alexa Fluor 488, Molecular Probes, USA, 1:200 dilution) for 30 min in the dark. Oocytes were washed in PBS one last time and transferred to an object glass with a drop of Vectashield Mounting Medium with DAPI (Vector, USA) for 15 min in the dark. The negative controls used similar conditions except for the substitution of primary antibodies with PBS.

Immunolabelling was observed under a fluorescence microscope (Nikon Eclipse E600, Nikon Inc., Japan) and confocal microscope (Zeiss LSM900, Carl Zeiss AG, Germany). The images obtained with the confocal microscope were further processed with Zeiss ZEN software (Blue edition, Carl Zeiss AG, Germany) to prepare 3D reconstructions.

Analysis of total cell fluorescence by fluorescence microscopy (Nikon Eclipse E600) was performed for the comparison of the expression level of AMHR2 protein using Fiji software [40]. Total cell fluorescence by fluorescence microscopy was calculated with the help of an adjusted formula found in Gavet and Pines [41]:

Corrected total cell fluorescence (CTCF) = whole-cell fluorescence – (area selected x mean fluorescence of background readings).

For each image, we measured the fluorescence of the oocyte and the background (separately) in three repetitions. The average of each cell and its background was then used for the calculation of CTCF. CTCF is presented as average fluorescence ± SD.

In addition, a western blot analysis was performed to test the specificity and validity of the anti-AMHR2 antibody used in this study (Ab64762, Abcam, USA, 1:100 dilution) compared to the new primary polyclonal rabbit anti-AMHR2 antibody of the same producer (Ab197148, Abcam, USA, 1:100 dilution). With this analysis, we have confirmed the specificity and validity of the antibody Ab64762. The comparative western blot image with the strongest band at 63 kDa corresponding to AMHR2 detected by both antibodies is shown in Additional file 1.

### In vitro maturation of immature oocytes

Ninety-one immature (GV) oocytes from 45 patients were matured using the MediCult IVM system (LAG and IVM medium, Cooper Surgical, Denmark). Briefly, after acquiring denuded GV oocytes without surrounding follicular cells, they were transferred to LAG medium for 2 h. Later, cells were transferred to the 9-microwell culture dish (Vitrolife, Sweden) with one of four different IVM media applied: (1) IVM medium with the addition of recombinant AMH 100 ng/mL (1737-MS-010, R&D Systems, USA; *n* = 15 oocytes), (2) IVM medium with the addition of FSH 75 mIU/mL (Gonal-F, Merck Europe B.V., The Netherlands; *n* = 22 oocytes) and hCG 100 mIU/mL (Ovitrelle, Merck Europe B.V., The Netherlands), (3) IVM medium with the addition of FSH 75 mIU/mL, hCG 100 mIU/mL and AMH 100 ng/mL (*n* = 42 oocytes) and (4) IVM medium with no added hormones (control; *n* = 12 oocytes). The cells were then incubated in a CO_2_ incubator (37.0 °C and 6% CO_2_ in air) with the PrimoVision time-lapse microscope (Vitrolife, Sweden) to follow the maturation of the oocyte. Oocytes were observed until their maturation (extrusion of a polar body) for a maximum of 28 h. The time of GV breakdown and extrusion of the polar body (MII) was assessed using videos of maturation provided by time-lapse microscopy. All matured oocytes were frozen for later RT–qPCR analysis, while nonmatured oocytes were discarded.

### Single-cell RT–qPCR

RT–qPCR analysis of the *AMHR2* gene was performed using the Single Cell-to-CT kit (Ambion, USA). The kit includes solutions for single-cell lysis, reverse transcription, preamplification and real-time (q) PCR. TaqMan gene expression assays were used for gene expression analysis (Hs01086646-g1 for *AMHR2*, Hs00824723_m1 for *UBC*, Hs02758991-g1 for *GAPDH* and Hs99999909_m1 for *HPRT1*, Thermo Fisher Scientific, USA). The *UBC*, *GAPDH* and *HPRT1* genes were used as reference genes. However, after the completion of RT–qPCR, only *UBC* was used as an optimal reference gene for the obtained results, as chosen by Normfinder software [42]. qPCR was performed in a 384-well plate on a LightCycler 480 System (Roche Diagnostics GmbH, Germany) as described in the kit and TaqMan gene expression assays manual. The analysis was performed in 15 groups of oocytes with 5 oocytes per group; 9 groups consisted of oocytes that matured in vitro in three different IVM media with added hormones, 3 groups of immature (GV) oocytes and 3 groups of oocytes that were retrieved as mature oocytes by follicular aspiration but did not fertilize in the IVF programme. Additionally, each sample was analysed in triplicate. Differential expression analysis was performed using the comparative quantification algorithm -ΔΔCt [43].

### Statistical analysis

Statistical analysis was performed using SPSS software (IBM Corp., USA), GraphPad Prism software (GraphPad Software, USA) and Jamovi software [44]. The results obtained by immunocytochemistry and oocyte maturation in vitro were evaluated by Student’s t-test or Fisher’s exact test, while gene expression results obtained by RT–qPCR analysis were evaluated by Kruskal–Wallis one-way analysis of variance. A *P*-value < 0.05 was regarded as statistically significant.

## Results

### Patient information

There were no significant differences in the age, body mass index (BMI), serum FSH or luteinizing hormone (LH) levels among women donating oocytes for different analyses or for different oocyte groups for IVM with different maturation media and analysis of gene expression, as shown in Additional files 2 and 3.

### Detection of AMHR2 protein in immature and mature human oocytes with fluorescence and confocal microscopy

**Fig. 1:**
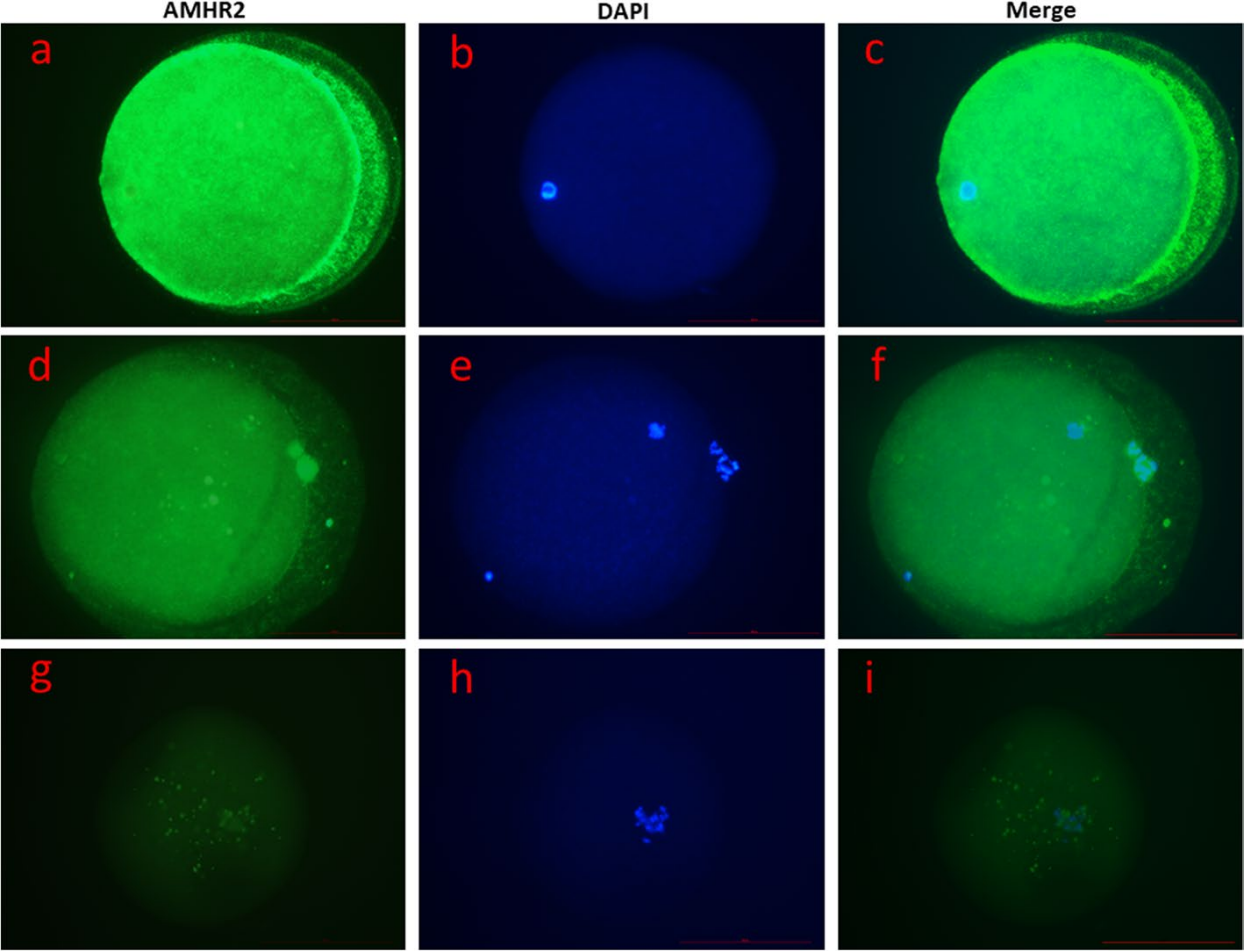
Expression of AMHR2 protein (green) in human oocytes, as revealed by immunocytochemistry. **A-C** Immature oocyte with germinal vesicle (GV). **D-F** Mature (MII) oocyte. **G-I** Control immature oocyte with germinal vesicle (primary antibody omitted). Images were taken with a fluorescence microscope at 400 × magnification, and the scale bar represents 100 µm. AMHR2 protein is stained green, and genetic material is stained blue (DAPI)

**Fig. 2:**
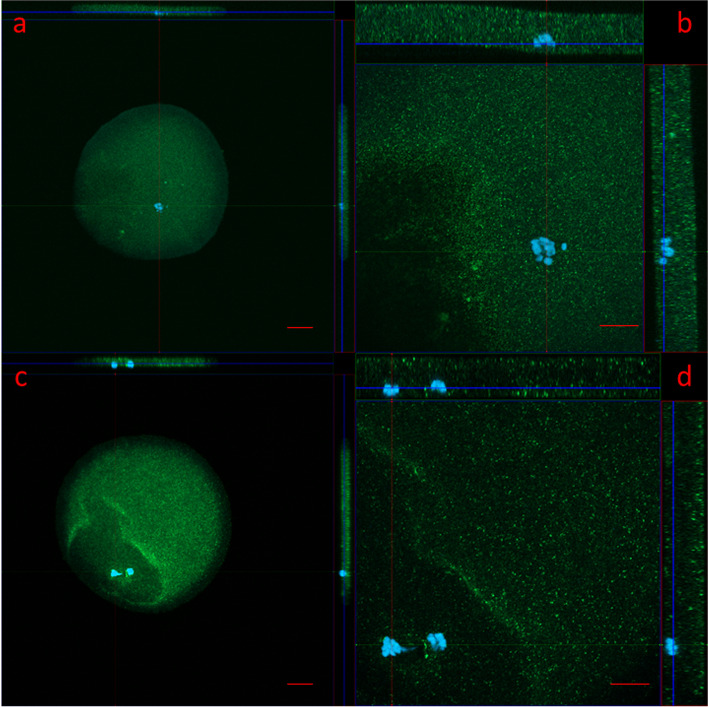
Expression of AMHR2 protein (green) in human oocytes, as revealed by confocal microscopy. **A** Immature oocyte with a germinal vesicle (blue). **B** Enlarged section of the same immature oocyte with side horizontal cuts showing expression of AMHR2 protein throughout the oocyte. **C** Mature oocyte with a polar body. **D** Enlarged section of the same mature oocyte with side horizontal cuts showing expression of AMHR2 protein throughout the oocyte. Images were taken with a confocal microscope at 200 × (images on the left, scale bar represents 20 µm) and 630 × (images on the right, scale bar represents 10 µm) magnification. AMHR2 protein is stained green, and genetic material is stained blue (DAPI). Blue lines show the position of genetic material in the horizontal cuts of oocytes from above or from the side

**Fig. 3:**
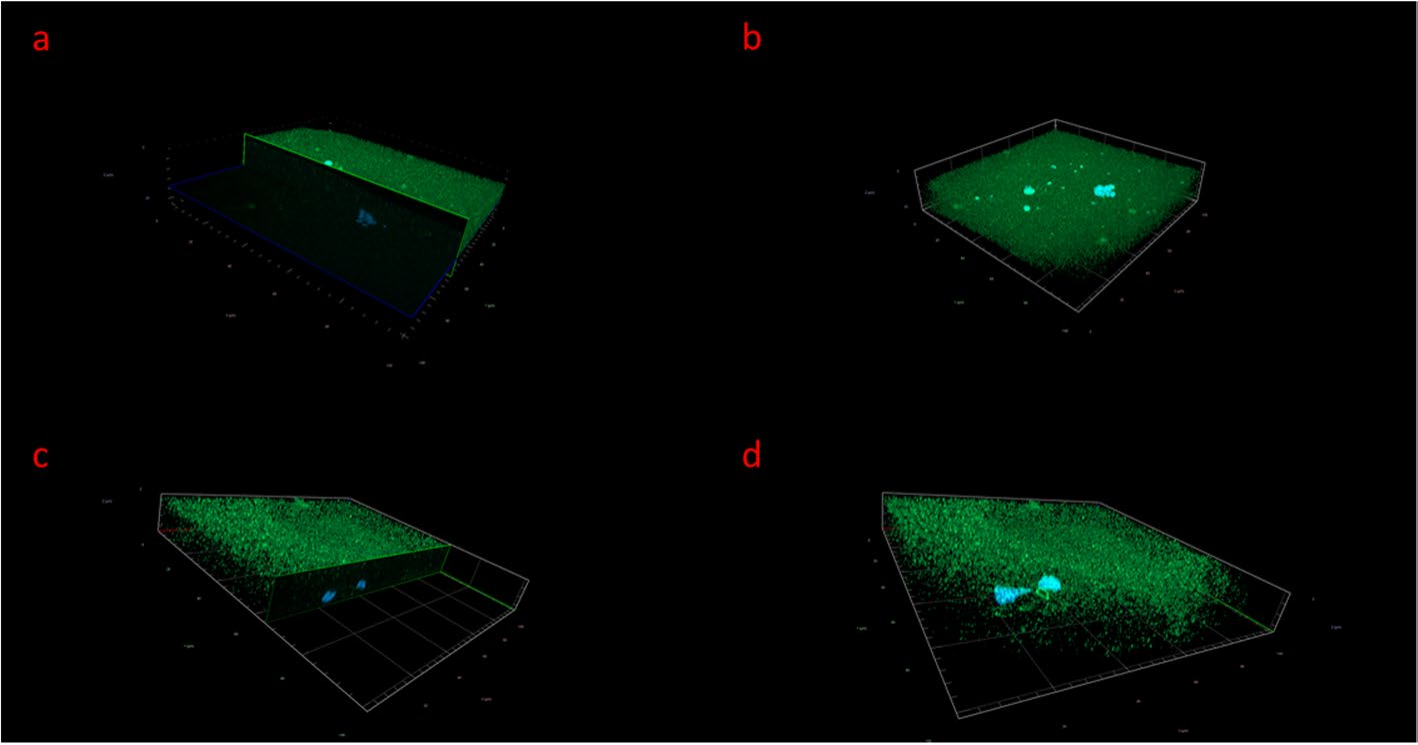
Expression of AMHR2 protein (green) in human oocytes, as revealed by confocal microscopy. **A** Horizontal section of an AMHR2-positive immature oocytes. **B** Immature oocyte positively stained for AMHR2 protein. **C** Horizontal section of an AMHR2-positive mature oocyte. **D** Mature oocyte positively stained for AMHR2 protein. Images were taken with a confocal microscope at 630 × magnification. AMHR2 is stained green, and genetic material is stained blue (DAPI)

Of 126 oocytes (65 GV, 61 MII) collected for immunocytochemistry, 59 oocytes were successfully stained (33 GV, 26 MII; 2 GV oocytes from the GV pool were used as control). We observed the expression of AMHR2 protein on the surfaces and inside of both mature and immature oocytes (Figs. 1, 2, 3, Additional file 4). The differences in expression between these two groups of oocytes (8.1 × 10^7^ ± 3.4 × 10^7^ CTCF vs. 7.7 × 10^7^ ± 3.0 × 10^7^ CTFC) were not statistically significant, as revealed by Student’s t-test. The data is presented as average fluorescence ± SD. However, we observed that AMHR2 protein was expressed in a spotted pattern throughout the whole cell (Fig. 3). Conversely, in the negative control, the expression of AMHR2 protein was almost not visible (Figs. 1g and 1i).

Videos of AMHR2-positive oocytes stained by immunocytochemistry and recorded by confocal microscopy can be seen in the appendix (Additional file 5).

### Effect of AMH in medium on the maturation of GV oocytes in vitro

**Fig. 4:**
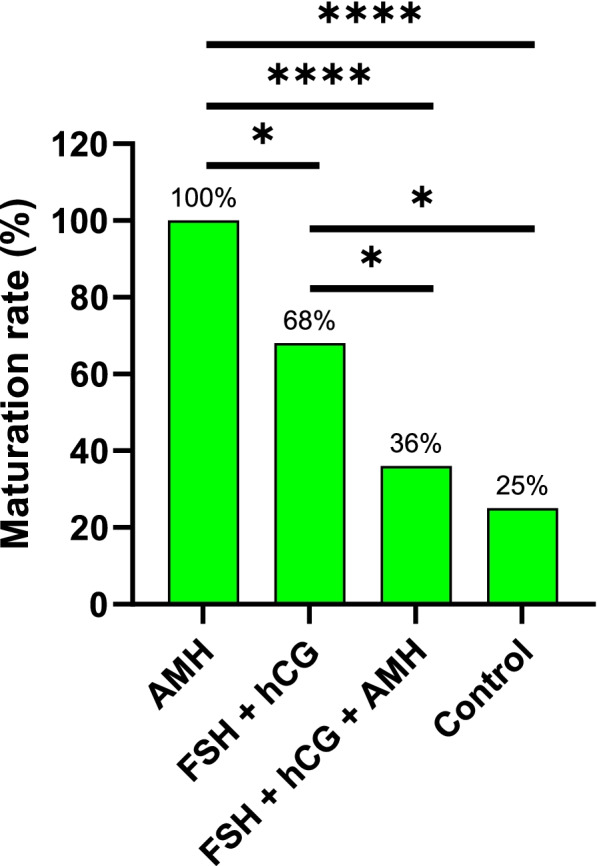
Maturation rates of GV oocytes matured in vitro. IVM medium containing one of the following combinations was used: i) AMH, ii) FSH + hCG, iii) FSH + hCG + AMH, or iv) no added hormones (control). Different levels of significance are indicated as follows: *P < 0.05 and ****P < 0.0001 (Fisher’s exact test)

After IVM, we observed a 100% (15/15) maturation rate of oocytes that matured in the medium with added recombinant AMH only. In comparison, only a 68% (15/22) maturation rate was achieved with the use of conventional IVM medium with added FSH and hCG. Next, the group of oocytes that were matured in IVM medium supplemented with all three hormones (FSH, hCG and AMH) produced an even lower maturation rate of 36% (15/42). Last, in the control group of oocytes that were matured in IVM medium without hormones added, 25% (3/12) of oocytes spontaneously matured in vitro (Fig. 4). All differences between groups, except between the last two groups, were statistically significant (Fisher’s exact test, *P* < 0.05), as shown in Fig. 4.

### Effect of AMH in IVM medium on the maturation dynamics of oocytes: GV breakdown and polar body release

**Fig. 5:**
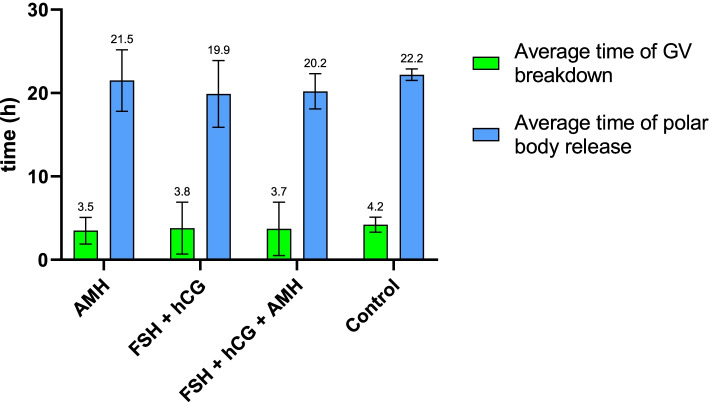
Average time (hours) to GV breakdown and polar body release in oocytes matured in vitro. IVM medium containing one of the following combinations was used: i) AMH, ii) FSH + hCG, iii) FSH + hCG + AMH, or iv) no added hormones (control). Data represents mean ± SD. There was no significant difference between the groups (Student’s t-test)

The mean times were 3.7 h to GV breakdown (MI stage) in oocytes and 20.5 h to polar body release (MII stage) (Fig. 5). The times needed to reach the MI stage were comparable among oocytes that were matured in the AMH, FSH + hCG, and FSH + hCG + AMH media and were 3.5, 3.8 and 3.7 h, respectively (Fig. 5). There was a tendency for the polar body to be released later if AMH was added to the IVM medium: 21.5 and 20.2 vs. 19.9 h (Fig. 5). However, the differences were not statistically significant (Student’s t-test, *P* < 0.05). In the control group of oocytes, both times were prolonged (4.2 and 22.2 h) due to their slower spontaneous maturation.

**Fig. 6:**
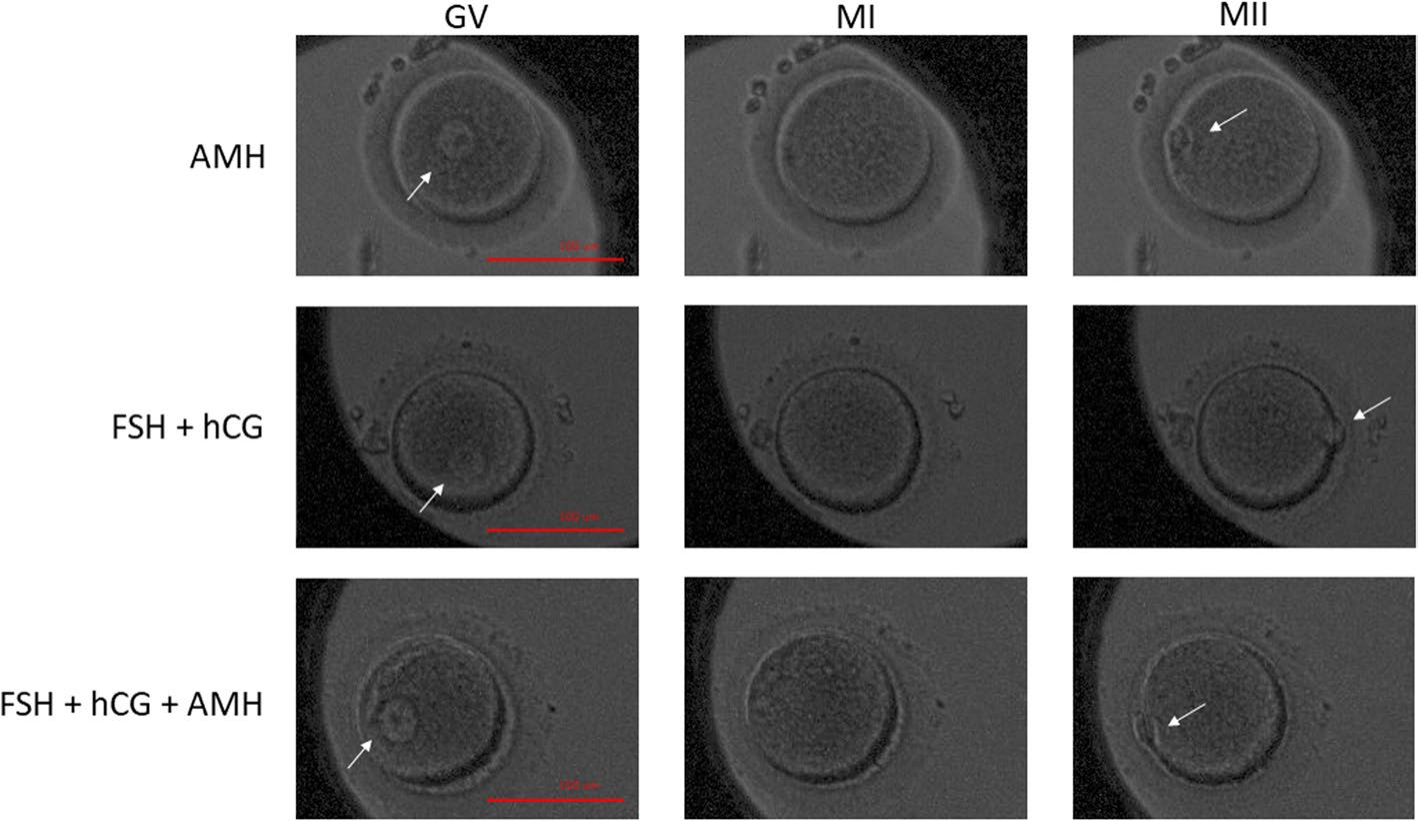
Non-fluorescent images of in vitro maturing human oocytes under different conditions, as revealed by time-lapse microscopy. IVM medium containing one of the following combinations of hormones was used: i) AMH, ii) FSH + hCG and iii) FSH + hCG + AMH. Legends: **GV**—Immature oocytes with germinal vesicle (arrow), **MI**—Immature oocytes at the MI stage, and **MII**—Mature oocytes at the MII stage with extruded polar body (arrow). All images were taken from the same oocytes at different time periods using PrimoVision time-lapse microscopy, the scale bar represents 100 µm

Figure 6 shows images of oocytes maturing in vitro in various media (AMH, FSH + hCG and FSH + hCG + AMH) under time-lapse microscopy.

### Expression of the *AMHR2* gene in immature and mature human oocytes

**Fig. 7:**
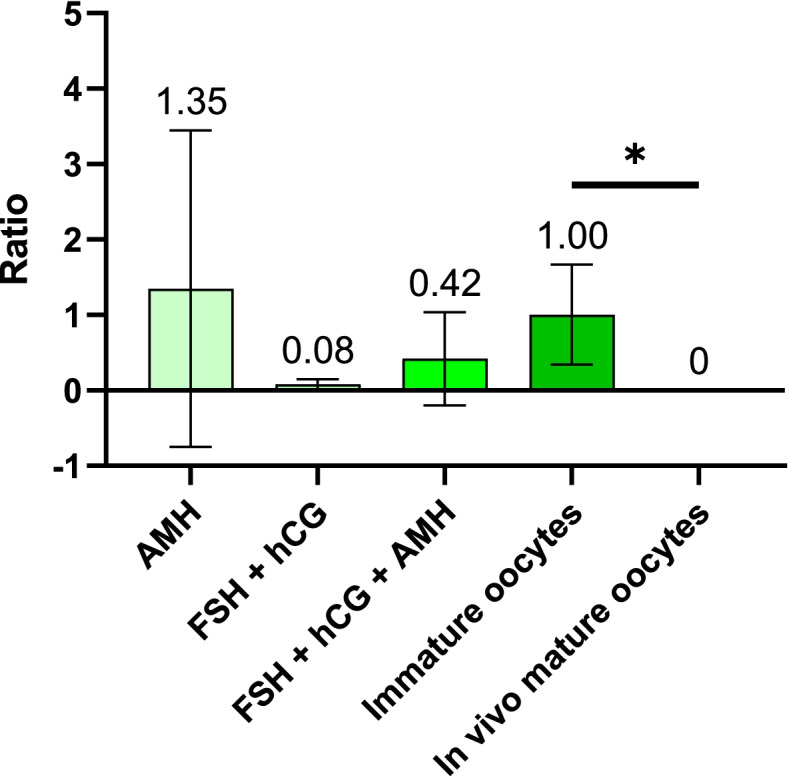
Average ratio of *AMHR2* gene expression in mature oocytes compared to immature oocytes. Five different groups are presented. The first three are in vitro matured oocytes in IVM medium supplemented with i) AMH, ii) FSH + hCG and iii) FSH + hCG + AMH. The last two groups represent untreated immature oocytes and in vivo mature oocytes. Data represents mean ± SD. Statistical significance is shown at *P < 0.05 (Kruskal–Wallis one-way analysis of variance)

RT–qPCR analysis confirmed the expression of the *AMHR2* gene in immature and in vitro matured oocytes (Fig. 7). The expression of this gene was highest in oocytes that matured in medium with only added AMH and in immature (GV) oocytes. The expression of the *AMHR2* gene was lower in oocytes that matured in the presence of all three hormones (AMH, FSH and hCG). Finally, the lowest expression was observed in the conventional mode of IVM with FSH and hCG added to the maturation medium. Nevertheless, the differences were not statistically significant (Kruskal–Wallis one-way analysis of variance, *P* < 0.05).

Additionally, we observed that the *AMHR2* gene was expressed in immature (GV) and in vitro matured oocytes, while it was not expressed in oocytes that matured in vivo. The difference between immature oocytes and in vivo mature oocytes was statistically significant (Kruskal–Wallis one-way analysis of variance, *P* < 0.05).

In Additional file 6 and 7, box plots and Q-Q plots of *AMHR2* gene expression in human oocytes obtained by RT–qPCR can be seen. The data comparing *AMHR2* gene expression in oocytes matured under different conditions are presented in Additional file 8.

## Discussion

The results of our study show for the first time that human oocytes express the protein and gene for AMHR2, the receptor for the hormone AMH. Moreover, in an IVM model, we found that AMH acts directly on oocytes and improves oocyte maturation in vitro.

In this study, in vitro matured oocytes were free of granulosa cells, which had been previously removed by oocyte denudation using hyaluronidase. It was confirmed that both AMHR2 protein and mRNA encoding it are expressed in human oocytes. Expressed mRNA along with the protein allows us to conclude that the oocytes probably synthesise AMHR2 on their own, which is a completely new finding.

AMHR2 protein was expressed in both immature and mature human oocytes, while the gene for this protein, *AMHR2,* was expressed only in immature and in vitro matured oocytes. We speculate that the *AMHR2* gene was expressed only in immature oocytes, as AMH is needed for their maturation. After maturation, AMHR2 activity is probably no longer required, so the expression of this gene is stopped. However, the AMHR2 protein is probably still expressed in oocytes, as the receptors are not removed or recycled by the cell after maturation. Regardless, further research is needed.

The AMRH2 protein is thought to be located in the cell membrane as it is a membrane receptor [35]. Despite the fact that it is supposed to be a surface antigen, we also performed permeabilization in the process of immunocytochemistry of oocytes. Permeabilization of the cell membrane is an extremely important step in detecting intracellular antigens with a primary antibody, because it allows entry across the cell membrane. Permeabilization is introduced after cells have been prepared with a fixative agent to initiate protein cross-linking. In our case, the fixative agent was paraformaldehyde. To permeabilize the membrane, we used a detergent Triton X-100 which permeabilizes the membrane by inserting a detergent monomer into the lipid membrane. This procedure was correct, as oocyte immunocytochemistry on AMHR2 was successfully performed. In addition, confocal microscopy in different planes showed that the AMHR2 protein is expressed throughout whole oocytes and not just on the surface as one would expect.

We performed IVM maturation of GV human oocytes under similar conditions to those of the study by Zhang et al. [35] in mouse oocytes. The addition of recombinant AMH alone to the IVM medium not only enabled immature oocytes to mature but also helped all the treated oocytes to mature (100% maturation rate). This indicates that AMH directly affects human oocyte maturation in vitro and even improves it. In our study, the positive effect of recombinant AMH on the maturation of immature human oocytes in vitro was even greater than that of the commonly used hormones for IVM maturation – FSH and hCG. Perhaps this new knowledge can be used to further enhance the success of human oocyte IVM in the future, as this may mean that the use of gonadotropins as a culture supplement is not needed or even not recommended in the procedure of IVM. Further research is needed with a higher number of oocytes, especially to test different concentrations of AMH added to the maturation medium, as well as to answer the question of whether maturation using recombinant AMH has an effect on the (epi)genetic status of oocytes.

AMH and FSH play antagonistic roles in human folliculogenesis [37, 38]. In this study, we observed that the addition of AMH, FSH and hCG to the maturation medium had a negative effect on oocyte maturation, which was significantly lower than that of IVM with AMH alone or FSH plus hCG. In contrast, Zhang et al. [35] used FSH and AMH together in IVM medium with no adverse effect on the maturation of mouse oocytes. Due to our findings, as also mentioned by certain researchers [37, 38], FSH and AMH have antagonistic actions and should not be used together in a maturation medium. It cannot be excluded that AMH and FSH have a different coaction depending on the species that is examined in vitro.

In comparison to Zhang et al. [35], we did not perform analysis on the expression of AMH in the cumulus cells as they were not the focus of this study and were removed during the oocyte preparation step. Despite this, we do agree that it is an important factor in trying to completely understand the function of AMH on the ovarian follicles and oocytes. Again, further research needs to be done on this manner, as it has the potential to change the clinical application of IVM in IVF cycles.

There are certain limitations to our study. The main limitation is that the number of oocytes used was fairly low. This is always the case when working with human oocytes, as they are hard to obtain in higher numbers. The proportion of immature oocytes after controlled ovarian hormonal stimulation is relatively low (10–15% of all retrieved oocytes) [1, 2]. Additionally, some of the GV oocytes also matured spontaneously, did not mature in vitro or degenerated during immunocytochemistry and were not included in the study.

## Conclusions

In conclusion, we found that AMHR2 protein is expressed in both immature and mature human oocytes, which indicates that AMH can act on human oocytes directly. The *AMHR2* gene was expressed in immature and in vitro matured oocytes but not expressed in oocytes that matured in vivo in the ovaries. This further proves that AMH is important in the process of oocyte maturation, at least in vitro. Furthermore, the addition of only recombinant AMH to the maturation medium enabled and even improved the maturation of oocytes in vitro compared to the conventional IVM procedure with FSH and hCG in the maturation medium. The hormones AMH, FSH and hCG showed antagonism during oocyte maturation in vitro and should not be added to the maturation medium at the same time. Our findings prove that the current IVM protocol for immature human oocytes is not optimal and should be re-evaluated with the possibility of using recombinant AMH in the maturation medium.

## Supplementary Information

**Additional file 1.** Western blot analysis of ascites cells from a patient with recurrent ovarian cancer for expression of AMHR2 protein, using old mouse (Ab64762) and new rabbit (Ab197148) anti-AMHR2 antibodies.

**Additional file 2.** Average age, BMI, FSH and LH levels of patients whose oocytes were used for IVM. Differences between groups were not statistically significant (ANOVA).

**Additional file 3.** The average age of donor women distributed between the groups of in vitro matured oocytes and immature or mature oocytes. There was no significant difference.

**Additional file 4.** Average cell fluorescence (ACF) measured in different groups of oocytes: immature, mature and control oocytes.

**Additional file 5.** A 360° video of a mature human oocyte that is positively stained for AMHR2 protein (green); genetic material is stained blue (DAPI). The video was generated using the confocal microscope.

**Additional file 6.** Box plot of RT–qPCR results for *AMHR2 *gene expression. Different groups of in vitro matured oocytes are represented with the median line and standard deviation.

**Additional file 7.** Q-Q plot of RT–qPCR results for *AMHR2 *gene expression. These plots show that all of the obtained results are fairly normally distributed despite the low number of samples. The outlier is the last group of in vivo matured oocytes, where no *AMHR2 *gene expression was observed. Because of this group, ANOVA was not possible, and Kruskal–Wallis one-way analysis of variance had to be used.

**Additional file 8.** Comparisons of oocyte groups for *AMHR2 *gene expression. Kruskal–Wallis one-way analysis of variance was performed. There was a statistically significant difference in *AMHR2 *gene expression between the control groups (MII control vs. GV control, in yellow). Statistical significance was set at *P* < 0.05.

## Data Availability

The datasets used and analysed during the current study are available from the corresponding author on reasonable request.

## Abbreviations

AMH: Anti-Müllerian hormone
AMHR2: Anti-Müllerian hormone receptor 2
ART: Assisted reproductive technology
BMI: Body mass index
COCs: Cumulus-oocyte complexes
CTCF: Corrected total cell fluorescence
FBS: Foetal bovine serum
FSH: Follicle-stimulating hormone
GnRH: Gonadotropin-releasing hormone
GV: Germinal vesicle
hCG: Human chorionic gonadotropin
ICSI: Intracytoplasmic sperm injection
IVF: In vitro fertilization
IVM: In vitro maturation
LH: Luteinizing hormone
OHSS: Ovarian hyperstimulation syndrome
PBS: Phosphate-buffered saline
PCOS: Polycystic ovaries syndrome
PGD: Preimplantation genetic diagnosis
POI: Primary ovarian insufficiency
RT–qPCR: Reverse-transcription quantitative real-time polymerase chain reaction
