# Supplementary figures and images for: Recombinant anti-Müllerian hormone in the maturation medium improves the in vitro maturation of human immature (GV) oocytes after controlled ovarian hormonal stimulation
Source: Reprod Biol Endocrinol. 2022 Jan 24;20:18. doi: 10.1186/s12958-022-00895-5 (PMC8785574; doi:10.1186/s12958-022-00895-5)

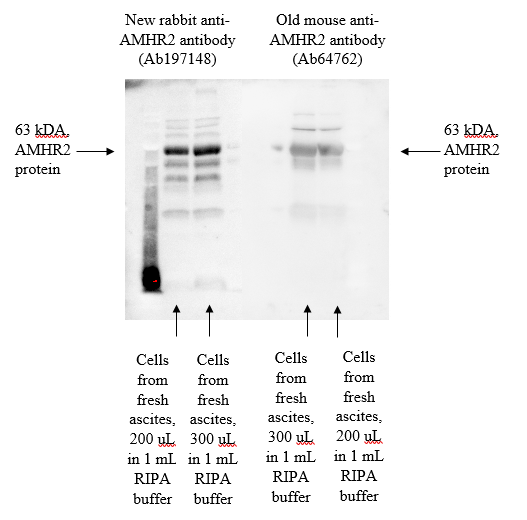

Supplement: Supplementary file 1 — Additional file 1. Western blot analysis of ascites cells from a patient with recurrent ovarian cancer for expression of AMHR2 protein, using old mouse (Ab64762) and new rabbit (Ab197148) anti-AMHR2 antibodies. [file 12958_2022_895_MOESM1_ESM.tif]

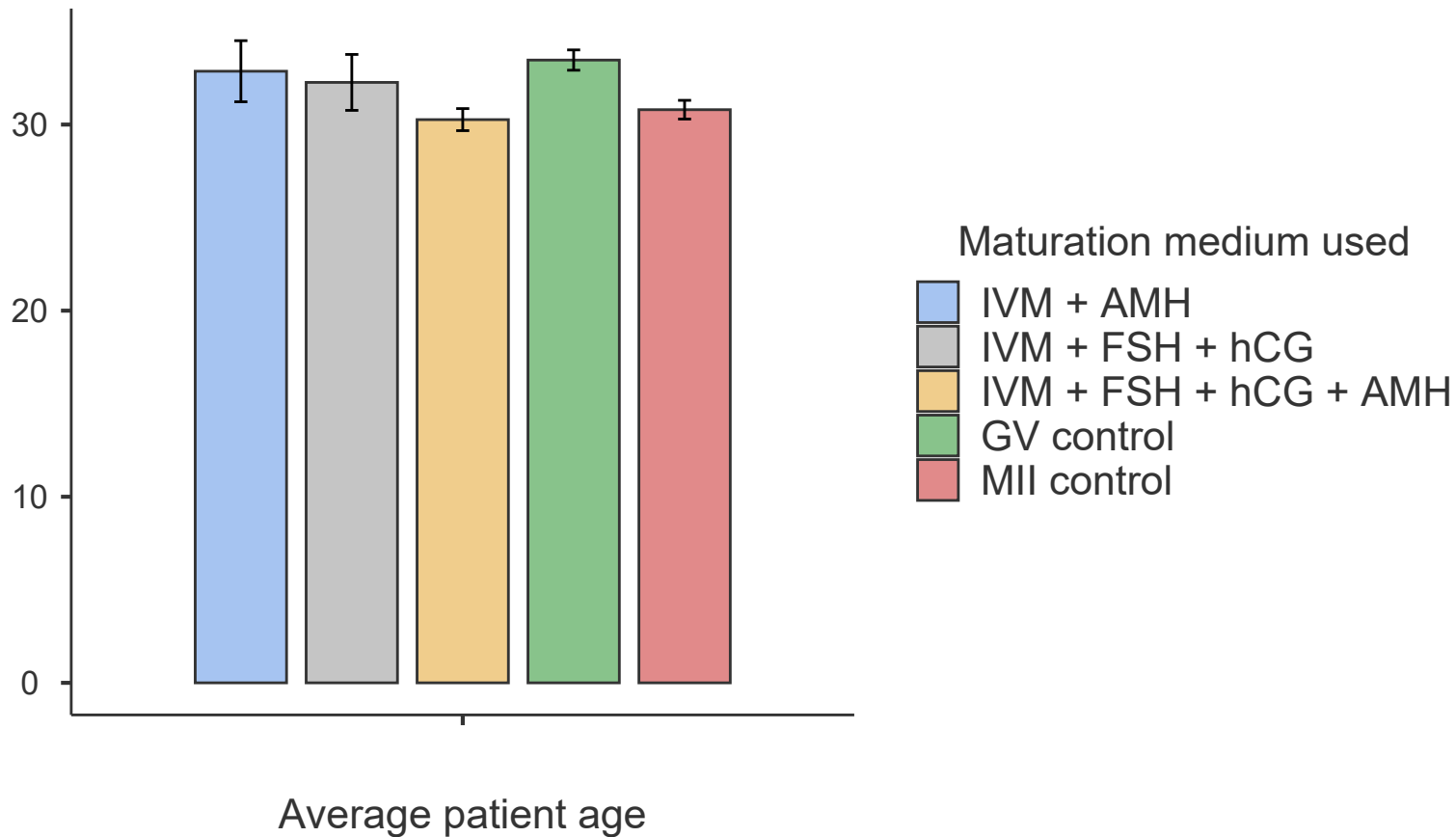

Supplement: Supplementary file 3 — Additional file 3. The average age of donor women distributed between the groups of in vitro matured oocytes and immature or mature oocytes. There was no significant difference. [file 12958_2022_895_MOESM3_ESM.pdf]

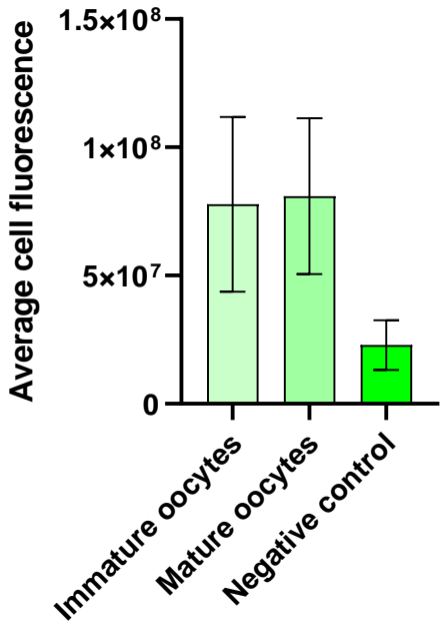

Supplement: Supplementary file 4 — Additional file 4. Average cell fluorescence (ACF) measured in different groups of oocytes: immature, mature and control oocytes. [file 12958_2022_895_MOESM4_ESM.pdf]

## Slide 1
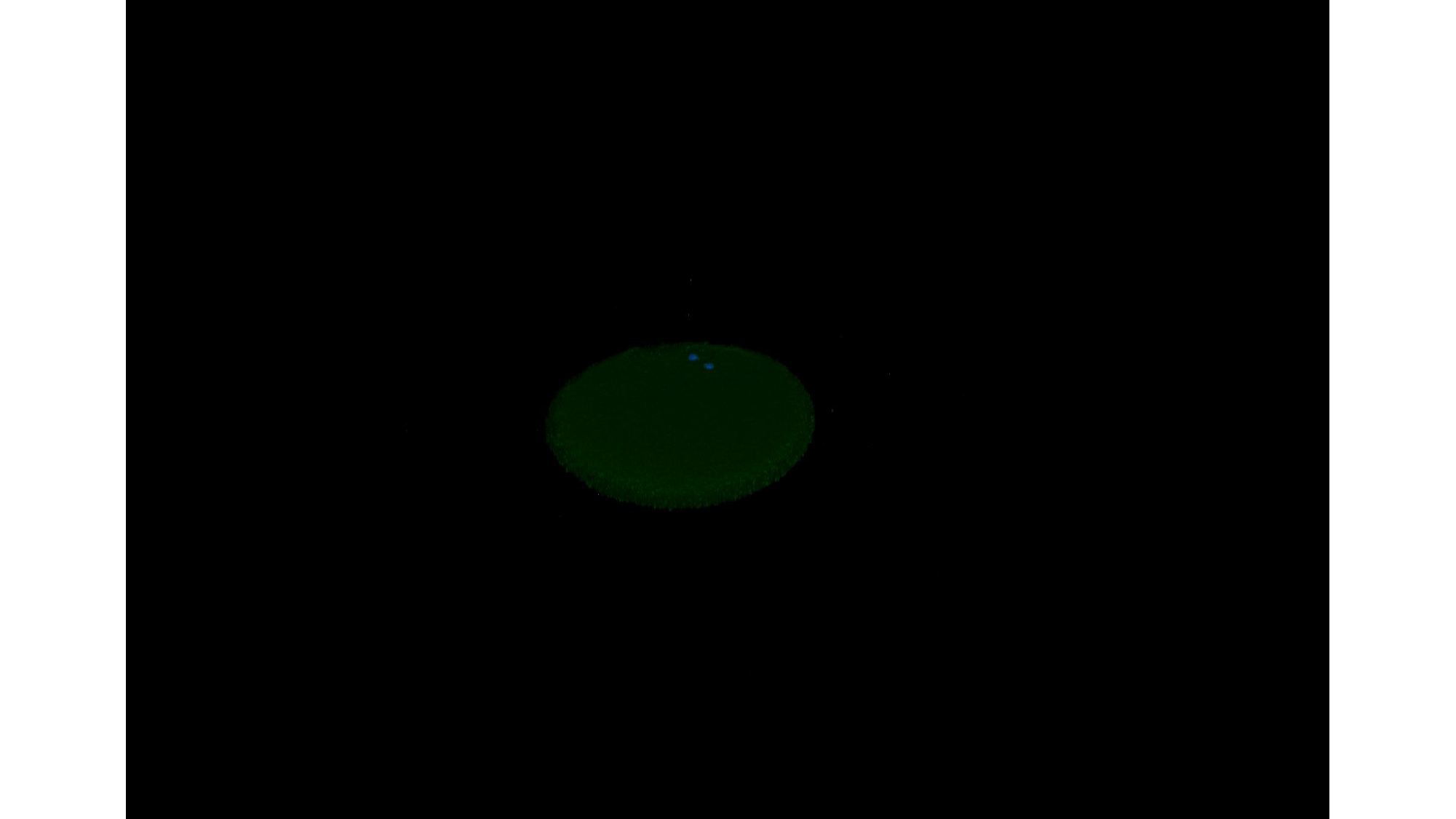

Supplement: Supplementary file 5 — Additional file 5. A 360° video of a mature human oocyte that is positively stained for AMHR2 protein (green); genetic material is stained blue (DAPI). The video was generated using the confocal microscope. [file 12958_2022_895_MOESM5_ESM.pptx]

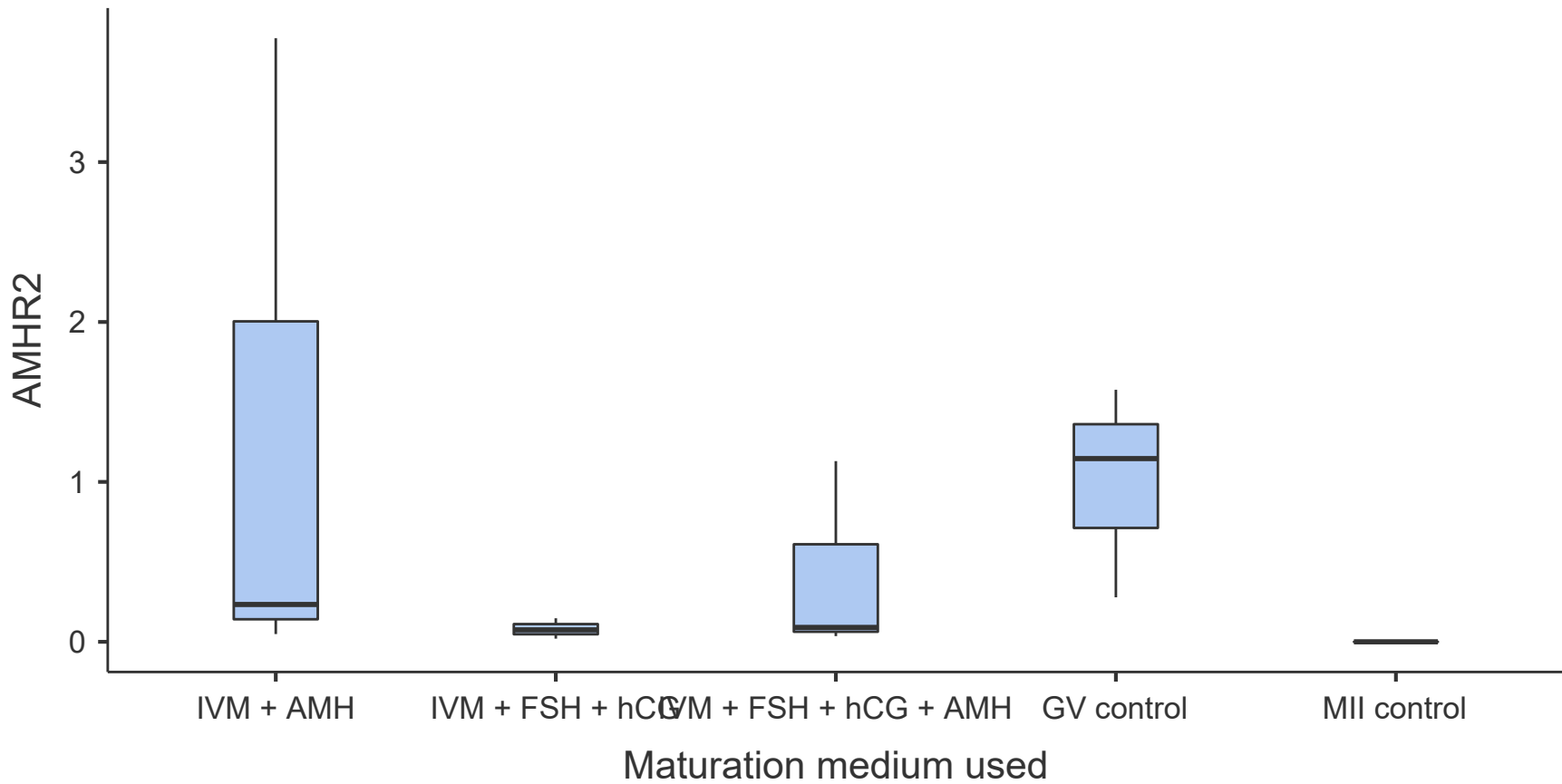

Supplement: Supplementary file 6 — Additional file 6. Box plot of RT–qPCR results for AMHR2 gene expression. Different groups of in vitro matured oocytes are represented with the median line and standard deviation. [file 12958_2022_895_MOESM6_ESM.pdf]

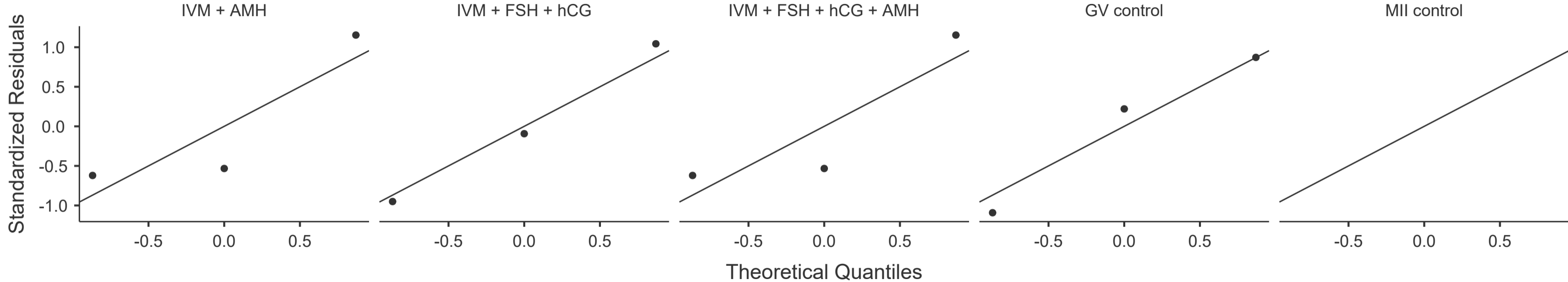

Supplement: Supplementary file 7 — Additional file 7. Q-Q plot of RT–qPCR results for AMHR2 gene expression. These plots show that all of the obtained results are fairly normally distributed despite the low number of samples. The outlier is the last group of in vivo matured oocytes, where no AMHR2 gene expression was observed. Because of this group, ANOVA was not possible, and Kruskal–Wallis one-way analysis of variance had to be used. [file 12958_2022_895_MOESM7_ESM.pdf]

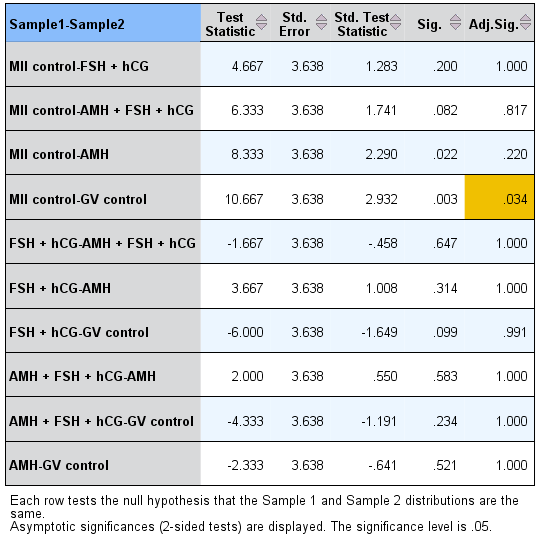

Supplement: Supplementary file 8 — Additional file 8. Comparisons of oocyte groups for AMHR2 gene expression. Kruskal–Wallis one-way analysis of variance was performed. There was a statistically significant difference in AMHR2 gene expression between the control groups (MII control vs. GV control, in yellow). Statistical significance was set at P < 0.05. [file 12958_2022_895_MOESM8_ESM.tif]
