# Supplementary material for: Recombinant anti-Müllerian hormone in the maturation medium improves the in vitro maturation of human immature (GV) oocytes after controlled ovarian hormonal stimulation
Source: Reprod Biol Endocrinol. 2022 Jan 24;20:18. doi: 10.1186/s12958-022-00895-5 (PMC8785574; doi:10.1186/s12958-022-00895-5)
Supplement: Supplementary file 2 — Additional file 2. Average age, BMI, FSH and LH levels of patients whose oocytes were used for IVM. Differences between groups were not statistically significant (ANOVA). [file 12958_2022_895_MOESM2_ESM.docx]

|  | **Age** | **BMI** | **FSH levels** | **LH levels** |
| --- | --- | --- | --- | --- |
| AMH | 32.9 ± 2.8 | 24.3 ± 1.7 | 5.9 ± 1.6 | 5.9 ± 0.9 |
| FSH + hCG | 32.3 ± 2.6 | 25.0 ± 2.0 | 5.7 ± 1.4 | 3.6 ± 0.6 |
| FSH + hCG + AMH | 30.3 ± 1.0 | 23.8 ± 1.0 | 5.8 ± 1.3 | 4.6 ± 0.8 |
| Immature oocytes | 33.5 ± 0.9 | 28.0 ± 3.1 | 7.5 ± 1.9 | 6.1 ± 0.9 |
| In vivo matured oocytes | 30.8 ± 0.9 | 27.3 ± 2.5 | 6.7 ± 1.1 | 5.6 ± 0.8 |
